# A Hybrid RF/FSO Transmission System Based on a Shared Transmitter

**DOI:** 10.3390/s25072021

**Published:** 2025-03-24

**Authors:** Qinyi Zhang, Jianjun Yu, Jianyu Long, Chen Wang, Jiali Chen, Xin Lu

**Affiliations:** State Key Laboratory of ASIC and System, Key Laboratory for Information Science of Electromagnetic Waves (MoE), School of Information Science and Technology, Fudan University, Shanghai 200433, China; qinyizhang23@m.fudan.edu.cn (Q.Z.); jylong22@m.fudan.edu.cn (J.L.); 22110720077@m.fudan.edu.cn (C.W.); 23210720141@m.fudan.edu.cn (J.C.); 23210720218@m.fudan.edu.cn (X.L.)

**Keywords:** hybrid transmission, shared transmitter, radio frequency, millimeter wave communication, free space optical, digital signal processing

## Abstract

In this work, we propose a novel design of a hybrid transmission integrated system for radio frequency (RF) and free-space optical (FSO) communications, in which the RF and FSO links are able to share the transmitter and the transmission link. In this system, the RF link can usually be considered as a complement to the FSO link, and the hybridization of the two transmissions allows the system to adapt to a variety of complex environments while maintaining a high rate of transmission, improves system stability, and greatly reduces system complexity through the design of a shared transmitter. An experimental demonstration of the system has been carried out, and the results show that the hybrid RF/FSO system supports 50 Gbit/s transmission, satisfying the 20% soft-decision forward error correction (SD-FEC) threshold of 2.4 × 10^−2^. Additionally, for the FSO link, the system supports 100 GBaud QPSK and 32 GBaud 16QAM transmissions individually. The proposed structure combines the advantages of RF and FSO, providing a foundation for future high-speed, broadband, all-environment communication.

## 1. Introduction

Free-space optical (FSO) communication serves as a critical enabler for applications demanding ultra-high-speed, secure, and reliable wide-bandwidth data transmission, including satellite-ground links, inter-satellite networks, and next-generation wireless access systems. This technology offers distinct advantages: access to unlicensed spectrum, exceptional spectral efficiency, highly directional beam propagation, and minimal signal attenuation under clear atmospheric conditions. The narrow beam divergence inherent in point-to-point FSO links inherently mitigates multipath interference, thereby simplifying carrier recovery algorithms in receiver-side digital signal processing (DSP). However, FSO performance is fundamentally constrained by atmospheric turbulence and meteorological impairments such as fog, haze, and snow, which induce severe range limitations and signal degradation [1,2,3]. Experimental measurements reveal attenuation exceeding 400 dB/km in dense fog conditions with visibility below 50 m [4]. Furthermore, FSO systems exhibit acute sensitivity to pointing errors, necessitating sub-millimeter-level alignment precision between transceivers to maintain line-of-sight propagation. Even minor mechanical vibrations or thermal drifts can disrupt optical path integrity, compromising link stability. Although state-of-the-art implementations have demonstrated 10 Tb/s over 220 m and 100 Gb/s over 1 km in controlled environments, persistent challenges from atmospheric disturbances continue to hinder the scalability and reliability of FSO technology [5].

As a robust complement to FSO systems, radio frequency (RF) links, particularly millimeter-wave (MMW) bands, demonstrate superior stability under adverse meteorological conditions. Field trials reveal that 40 GHz RF augmentation elevates system availability from 0.51% to 100% through hybrid signal diversity [6]. Unlike FSO’s stringent line-of-sight requirements, mmWave signals exhibit multipath resilience through reflection and diffraction mechanisms, enabling limited non-line-of-sight (NLOS) communication critical for urban environments with dense obstructions. While atmospheric turbulence and pointing errors constrain FSO to sub-kilometer operational ranges, mmWave systems achieve extended reach exceeding 30 km through adaptive beamforming and atmospheric penetration capabilities [7,8,9]. Therefore, integrating RF signals with FSO signals for transmission effectively combines the strengths of both communication methods, addressing the limitations inherent to single-mode communication in diverse scenarios while enhancing overall system bandwidth.

Hybrid RF/FSO systems are recognized as promising solutions for high-speed, point-to-point terrestrial communication in last-mile access networks, representing a pivotal approach for next-generation wireless networks beyond 5G [10,11,12]. This architecture offers a variety of potential application scenarios. For instance, in low Earth orbit (LEO) inter-satellite links (ISLs), Free Space Optical (FSO) communication provides high-speed data transmission, while Radio Frequency (RF) links serve as backup during satellite attitude adjustments or beam obstruction events. Additionally, the architecture can be applied to high-altitude platform stations (HAPS) and unmanned aerial vehicle (UAV) communication systems, using FSO for high-capacity backhaul links while relying on RF to maintain stable connectivity under adverse weather conditions that degrade optical signals [13,14]. For example, in extreme fog fading scenarios, RF links can maintain a base rate of 25 Gbps to ensure 100% availability, while FSO unleashes a high bandwidth potential of 425 Gbps in low-loss environments [15]. Meanwhile, the RF/FSO fusion transmission system is able to determine remote and efficient data transmission and sensing by combining FSO with radio frequency identification (RFID) sensing technology, which is particularly suitable for Internet of Things (IoT) and Industrial Internet of Things (IIoT) applications in complex environments [16,17,18,19]. Such systems not only optimize communication and sensing resources to improve reliability and coverage, but can also incorporate deep learning technologies to enhance signal processing capabilities, thus playing an important role in areas such as smart monitoring and structural health monitoring (SHM) [16,18].

**Figure 1:**
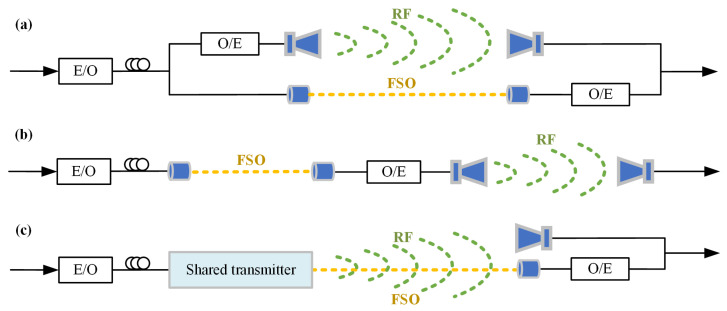
The hybrid RF/FSO system structure. (**a**) Discrete parallel structure; (**b**) discrete serial structure; (**c**) integrated shared transmitter structure. (Green: RF link; Yellow: FSO link).

For the transmission architecture, the proposed hybrid FSO/RF transmission models have been divided into two main categories. One of these is the serial transmission structure, where the FSO link is responsible for high-speed data transmission from the core network to remote base stations or relay nodes, leveraging its high capacity and immunity to spectrum interference. Meanwhile, in the access layer or complex environments, RF communication handles coverage from base stations to end-user devices, using its excellent multipath adaptability and wide-area flexibility to meet the demands of multiple users [20,21,22,23]. Another approach is to adopt a parallel architecture for FSO and RF transmission, where the system dynamically allocates resources for the RF and FSO links by monitoring real-time link quality (such as signal-to-noise ratio). Most parallel architectures are based on link selection and switching techniques, the FSO link carries primary traffic under high-quality conditions, while the RF link takes over or offloads data in low-quality scenarios, ensuring the continuity of service quality (QoS). This architecture is particularly well suited for high-speed backbone link deployment from the core network to remote base stations [12,24,25,26,27,28,29]. K. Wang et al. developed an RF/FSO hybrid fusion system that includes two FSO links and two RF links, achieving the transmission of an of a 1.196 Tbps signal over 800 m [30]. Moreover, a demonstration of hybrid fiber/FSO/millimeter wave transmission for 6G robust backhaul is also presented [31]. As shown in Figure 1, existing studies mainly use independent transmitters to realize RF and FSO converged systems, and the system requires two transmitters or two RF and FSO links, which increases the complexity and weight of the system. In addition, there are also studies from an algorithmic point of view, such as adaptive soft switching schemes based on machine learning [32] and neural network optical energy transmission optimization schemes [33]. From an antenna design perspective, Ref. [34] describes an FSO/RF composite antenna developed by M.M. Abadi et al., which achieves a common aperture for transmitting and receiving optical and RF signals. However, this design is bulky, structurally complex, and limited to data rates of 6.7 Mbps for FSO and 100 kbps for RF links. Subsequently, Ref. [35] presents an optimized FSO/RF antenna with a reduced size, but only tests 7 Mbps BPSK FSO signals. Furthermore, the integrated antenna structure does not support further amplification of fused signals post-generation.

In this paper, a hybrid RF/FSO integrated transmission architecture is proposed for the first time, which enables the system to transmit both RF and FSO signals on the same link by sharing the transmitter. Firstly, we discuss the hybrid RF/FSO transmission model, in which the attenuation suffered by each of the two links due to environmental influences is taken into account, and a proof-of-principle is then carried out to demonstrate the feasibility of such a shared transmitter architecture. The results show that the system supports the converged transmission of 25 GBaud RF/FSO signals, so such a shared transmitter and common link transmission model is feasible.

## 2. Principle

### 2.1. Design of Hybrid RF/FSO Structure

**Figure 2:**
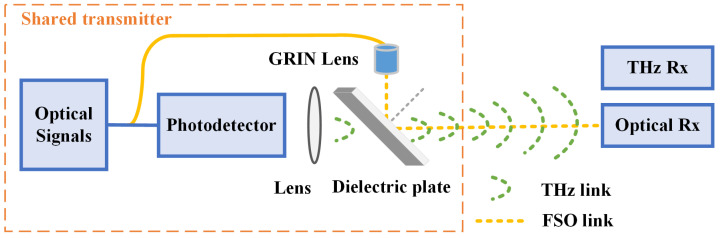
The structure of the hybrid RF/FSO system with a shared transmitter and transmission link.

As shown in Figure 2, the RF signals and optical signals are combined and transmitted through a structure comprising a dielectric plate, a gradient-index (GRIN) lens, and a lens for RF focusing. The dielectric plate serves to reflect optical signals and can be fabricated from optical plastics that are transparent to the millimeter-wave band and capable of reflecting light. Polymethyl methacrylate (PMMA), a widely used optical plastic, is an example of such a material. It is a polymer commonly known as Plexiglas and exhibits high optical reflectivity and MMW transparency. Measurements indicate that, for incident optical power of 14 dBm, PMMA transmits 12 dBm and reflects 2 dBm. In this study, we take advantage of its MMW high transmittance property by which RF signals can be transmitted without excessive loss, and its reflection property for optical signals by which FSO signals can propagate along the same channel, thus enabling a shared transmitter architecture. The GRIN lens, also referred to as a self-focusing lens, is employed for collimation and focusing of optical signals, producing a parallel beam after collimation. In this study, a Thorlabs F810FC-1550 GRIN lens (Newton, NJ, USA) was used, which operates at 1550 nm and can effectively reduce beam divergence and improve the transmission stability of the system. For the RF focusing lens, plano-convex TPX (Polymethyl pentene) aspheric lenses from Thorlabs are adopted, as they exhibit broadband transparency in the MMW range and effectively perform collimation and a focusing of MMW signals. Experimental findings confirm that incorporating a lens after the photodetector can provide gain, which is dependent on the lens dimensions.

After generating the optical signal at the transmitter, the signal is divided into two separate paths. In one path, a photodetector performs photoelectric conversion, and the resulting electrical signals are focused by a lens before passing through a dielectric plate and being emitted into free space. In the second path, the optical signals are transmitted through an optical fiber, with a GRIN lens used to create a parallel beam that is incident on a PMMA plate. The optical signals are then reflected by the dielectric plate, allowing them to travel along the same path as the electrical signals. The fusion signals are received by the respective RF and optical receivers. The optical receiver must be precisely aligned with the transmitter, while the electrical receiver can be positioned adjacent to the optical receiver. This structure enables the fusion transmission of RF and FSO signals through the shared transmitter.

To verify the feasibility of this novel fusion transmission scheme, we chose to conduct experiments in a stable, short-range indoor laboratory environment. In this setting, the effects of atmospheric turbulence, pointing errors, and scintillation in the FSO channel are negligible. Additionally, to account for system nonlinearities, we strictly controlled the input signal power to remain within the linear region of the devices. Upon receiving the signal, we employed a series of digital signal processing (DSP) algorithms for carrier recovery to achieve optimal bit error performance.

### 2.2. Channel Models

#### 2.2.1. RF Link

For the RF link in the converged transmission architecture, the received signal can be expressed as

(1) $$y_{R}=\sqrt{P_{R}h_{l,R}}\;h_{R}s+n_{R}$$

where *s* is the transmitted symbol with unit energy, $P_{R}$ is the transmit power of the RF link, and $h_{R}$ is the fading coefficient of the RF link, which follows the Nakagmi-m distribution. $n_{R}$ is the additive white Gaussian noise (AWGN) of the RF link with mean 0 and variance $\sigma_{R}^{2}$. Path loss $h_{l,R}$ is given as

(2) $$h_{l,R}=G_{t,R}+G_{r,R}-20lg\left(\frac{4\pi L_{R}}{\lambda_{R}}\right)-\left(\alpha_{\mathrm{oxy}}+\alpha_{\mathrm{rain}}\right)L_{R}$$

where $G_{t,R}$ and $G_{r,R}$ represent the gain of RF link transmitting antenna and receiving antenna, respectively, $L_{R}$ is the RF link distance, $\lambda_{R}$ is the RF link wavelength, and $\alpha_{\mathrm{oxy}}$ and $\alpha_{\mathrm{rain}}$ are the attenuation coefficient caused by oxygen and rain absorption, respectively.

Since the received instantaneous signal-to-noise ratio (SNR) by the RF link is expressed as $\gamma_{R}={\bar{\gamma }}_{R}{\left|h_{R}\right|}^{2}$ and the average SNR is ${\bar{\gamma }}_{R}=\frac{P_{R}h_{l,R}}{\sigma_{R}^{2}}$, the probability density function (PDF) of $\gamma_{R}$ can be expressed as [36]

$$f_{\gamma_{R}}\left(x\right)={\left(\frac{m}{{\bar{\gamma }}_{R}}\right)}^{m}\frac{x^{m-1}}{\Gamma (m)}\exp\left(-\frac{mx}{{\bar{\gamma }}_{R}}\right)$$

where $\Gamma \left(\cdot \right)$ represents the upper incomplete Gamma function, and *m* is the parameter of channel fading degree. Finally, the cumulative distribution function (CDF) of $\gamma_{R}$ is given as

$$F_{\gamma_{R}}\left(x\right)=\frac{1}{\Gamma (m)}\gamma \left(m,\frac{mx}{{\bar{\gamma }}_{R}}\right)$$

#### 2.2.2. FSO Link

When it comes to the FSO link, considering the system as a coherent detection system, the received signal can be represented as

(3) $$y_{F}=\sqrt{P_{F}\eta I_{l}I}s+n_{F},$$

where *s* is the transmitted symbol with unit energy, $P_{F}$ is the transmit power of the FSO link, and η is the coefficient of the optical-to-electrical conversion. $I_{l}$ is the atmospheric attenuation associated with the FSO link, $I=I_{a}I_{p}$ is the fading coefficient, $I_{a}$ is the fading due to atmospheric turbulence, and $I_{p}$ represents the pointing error fading coefficient. $n_{F}$ is the AWGN with mean 0 and variance $\sigma_{F}^{2}$. The instantaneous received SNR $\gamma_{F}$ at the FSO receiver is given as

(4) $$\gamma_{F}=\frac{P_{F}\eta I_{l}I}{\sigma_{F}^{2}}.$$

According to the Beer–Lambert law, atmospheric attenuation $I_{l}=exp\left(-\sigma_{l}L_{F}\right)$, where $\sigma_{l}$ is the attenuation coefficient and $L_{F}$ is the transmission distance of the FSO link.

The atmospheric turbulence $I_{a}$ follows a Gamma–Gamma distribution, and the PDF is given as [37]

(5) $$f_{I_{a}}\left(x\right)=\frac{2{\left(\alpha_{F}\beta_{F}\right)}^{\frac{\alpha_{F}+\beta_{F}}{2}}}{\Gamma \left(\alpha_{F}\right)\Gamma \left(\beta_{F}\right)}x^{\frac{\alpha_{F}+\beta_{F}}{2}-1}\;K_{\alpha_{F}-\beta_{F}}\left(2\sqrt{\alpha_{F}\beta_{F}x}\right),$$

where $\alpha_{F}$ and $\beta_{F}$ is the fading coefficients of the atmospheric turbulence, and $K_{v}\left(\cdot \right)$ represents the $v^{th}$ order modified Bessel function of second kind.

As for the pointing error fading coefficient $I_{p}$, the PDF of $I_{p}$ can be expressed as [37]

(6) $$f_{I_{p}}\left(x\right)=\frac{\xi_{F}^{2}}{A_{0,\;F}^{\xi_{2}^{2}}}x^{\xi_{F}^{2}-1},0\leq x\leq A_{0,F},$$

where $\xi_{F}$ is the pointing errors parameter, and $A_{0,F}$ is the fraction of the collected power without any pointing error. Therefore, taking the above effects into account, δ is the average received power of the FSO link, the PDF of the FSO link’s SNR can be expressed as

(7) $$f_{\gamma_{F}}\left(\gamma_{F}\right)=\frac{\xi_{F}^{2}}{\Gamma \left(\alpha_{F}\right)\Gamma \left(\beta_{F}\right)\gamma_{F}}G_{1,3}^{3,0}\left({\left.\frac{\alpha_{F}\beta_{F}\gamma_{F}}{A_{0,F}\delta }\right|}_{\xi_{F}^{2},\alpha_{F},\beta_{F}}^{\xi_{F}^{2}+1}\right).$$

where $G_{p,q}^{m,n}\left[\cdot \right]$ represents the Meijer-G function. Finally, the CDF of $\gamma_{F}$ is given as

(8) $$F_{\gamma_{F}}\left(\gamma_{F}\right)=\frac{\xi_{F}^{2}}{\Gamma \left(\alpha_{F}\right)\Gamma \left(\beta_{F}\right)}G_{2,4}^{3,1}\left({\left.\frac{\alpha_{F}\beta_{F}\gamma_{F}}{A_{0,F}\delta }\right|}_{\xi_{F}^{2},\alpha_{F},\beta_{F},0}^{1,\xi_{F}^{2}+1}\right).$$

#### 2.2.3. Hybrid RF/FSO Link

According to the hybrid system architecture diagram, the link with higher SNR should be selected for data transmission in the system to optimize communication quality, so the total SNR of the system can be expressed as the maximum SNR of the two links, which is the hybrid SNR $\gamma_{\mathrm{hybrid}\;}=\max\left(\gamma_{F},\gamma_{R}\right)$. This means that, when the signal attenuation is high on one link, the system can use the signal received on the other link, thus avoiding performance impairments.

Based on probabilistic theory, the channel statistical characteristics of the FSO and RF links are considered to be independent, and the hybrid CDF can be derived as [36,37]

(9) $$\begin{array}{cc} F_{\gamma_{\mathrm{hybrid}}}\left(\gamma \right)\; & =P\left(\gamma_{\mathrm{hybrid}}\leq \gamma \right)=P\left(max\left(\gamma_{F},\gamma_{R}\right)\leq \gamma \right)\\ \; & =P\left(\gamma_{F}\leq \gamma \right)\cdot P\left(\gamma_{R}\leq \gamma \right)\\ \; & =F_{\gamma_{F}}\left(\gamma \right)\cdot F_{\gamma_{R}}\left(\gamma \right)\leq \min\left(F_{\gamma_{F}}\left(\gamma \right),F_{\gamma_{R}}\left(\gamma \right)\right). \end{array}$$

In a communication system, F(γ) can be interpreted as the probability that the SNR is below a certain threshold γ, that is, the probability that a disruption occurs in the link. Based on the above equation, it is deduced that the outage probability of the converged system is lower than that of any single link, which indicates that the converged system will not be out of service as long as the signal quality of any one of the links is good enough, which improves the reliability of the system. According to the hybrid SNR $\gamma_{\mathrm{hybrid}\;}=\max\left(\gamma_{F},\gamma_{R}\right)$, the hybrid system BER theoretical model can be expressed as

(10) $$P_{BER}^{hybrid}=P\left(\gamma_{F}\geq \gamma_{R}\right)\cdot P_{BER|F}\left(\gamma_{F}\right)+P\left(\gamma_{R}>\gamma_{F}\right)\cdot P_{BER|R}\left(\gamma_{R}\right),$$

so the hybrid BER can be expressed as

(11) $$\begin{array}{cc} P_{BER}^{hybrid} & =\int_{0}^{\infty }\left[P_{BER|F}\left(x\right)\cdot f_{\gamma_{F}}\left(x\right)\cdot F_{\gamma_{R}}\left(x\right)+P_{BER|R}\left(x\right)\cdot f_{\gamma_{R}}\left(x\right)\cdot F_{\gamma_{F}}\left(x\right)\right]\;dx\\ \; & \leq \int_{0}^{\infty }\min\left(P_{BER|F}\left(x\right),P_{BER|R}\left(x\right)\right)\cdot \left[f_{\gamma_{F}}\left(x\right)+f_{\gamma_{R}}\left(x\right)\right]\;dx\\ \; & =\min\left(E\left[P_{BER|F}\right],E\left[P_{BER|R}\right]\right). \end{array}$$

Therefore, the BER expectation of the system is always less than or equal to the minimum expectation of the BER of the two links. This inequality indicates that the fusion system, by dynamically selecting the low-BER link, strictly outperforms any single link.

## 3. Experimental Setup

**Figure 3:**
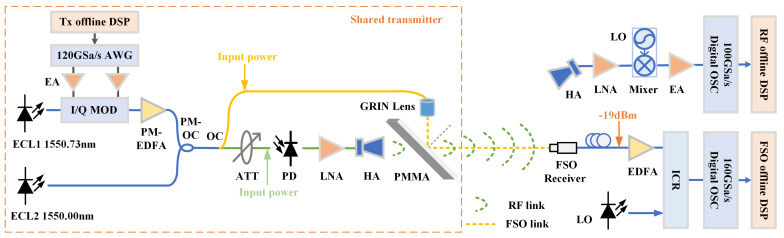
The experimental setup of the RF/FSO fusion system. (ECL: external cavity laser; DSP: digital signal processing; AWG: arbitrary waveform grating; I/Q Mod: I/Q modulator; PM-EDFA: polarization-maintaining Erbium-doped optical fiber amplifier; PM-OC: polarization maintaining optical coupler; OC: optical coupler; ATT: attenuator; PD: photodiode; LNA: low noise amplifier; HA: horn antenna; PMMA: Polymethyl methacrylate; FSO: free space optical; LO: local oscillator; EA: electrical amplifier; EDFA: Erbium doped fiber amplifier; ICR: integrated coherent receiver; OSC: oscilloscope.)

**Figure 4:**
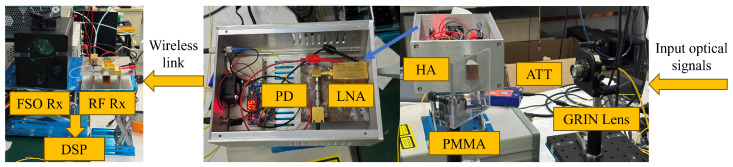
Key components of the shared transmitter physical diagram.

Figure 3 illustrates the experimental setup of the RF and FSO fusion system. On the transmitter side, a baseband signal is generated by a 120 GSa/s arbitrary waveform generator (AWG), model M8100 series from Keysight (Santa Rosa, CA, USA). The signal is then amplified by electric amplifiers (EAs) and modulated by an I/Q modulator onto an optical carrier generated by ECL1 (at 1550.73 nm). After passing through a polarization-maintaining Erbium-doped fiber amplifier (PM-EDFA) from Amonics (Hong Kong, China), the modulated signal is combined with the optical signal from ECL2 (at 1550.00 nm), resulting in a signal at 91.25 GHz, in the millimeter-wave band. The signal is split into two paths: in the electrical path, it first passes through an attenuator (ATT) to adjust the power input to the photodiode (PD), model Finisar XPDV3120R (Sunnyvale, CA, USA). The PD converts the optical signal into an electrical millimeter-wave signal, which then passes through a low-noise amplifier (LNA), model AT-LNA-75110-3504HP from AT Microwave (Shanghai, China) with 35 dB gain, and is transmitted via a horn antenna (HA) with 25 dBi gain into free space. The signal passes through a dielectric plate made of PMMA, transparent to MMW radiation. In the optical path, a self-focusing lens (GRIN lens), model F810FC-1550 from Thorlabs, generates parallel light incident on the PMMA plate, which is 5 mm thick. The plate reflects the optical signal, and by adjusting the angle of the plate, the optical and electrical signals remain on the same path. The wireless transmission distance in this experiment is 1 m, eliminating the need for lens-based focusing due to the short distance. The physical diagram of the key components in the shared transmitter is shown in Figure 4.

**Figure 5:**
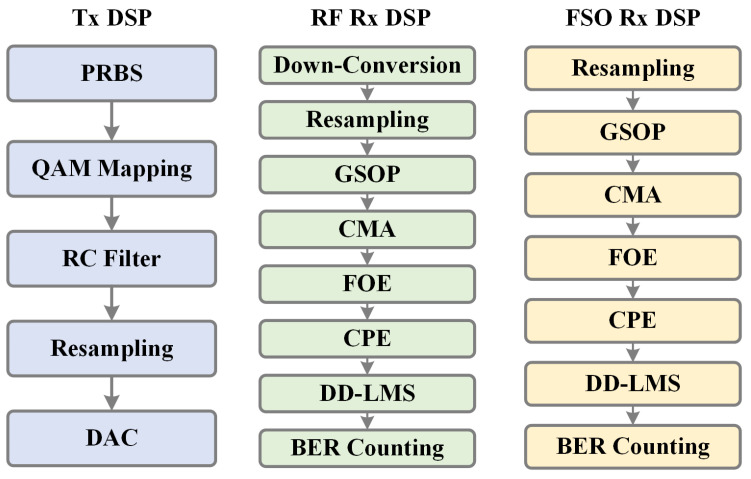
The digital signal processing (DSP) flowchart of the system.

At the receiving end, electrical and optical signals are simultaneously received by separate receivers. The optical receiver must be precisely aligned with the transmitter, while the electrical receiver can be positioned within a 0.2 m range from the optical receiver. For the FSO link, the optical signal received by the FSO receiver (model M1505009-02B, 34th Research Institute of China Electronics Technology Group Cooperation, CETC, Guilin, China) is pre-amplified by an Erbium-doped fiber amplifier (EDFA). Measurements indicate that, when the optical power input to the GRIN lens is 14 dBm, the power received by the fiber connected to the FSO receiver is −19 dBm, which is subsequently amplified by the EDFA. The signal is then processed by the integrated coherent receiver (ICR), with the local oscillator (LO) employed for homodyne detection. Finally, an oscilloscope (model DSA-X 96204Q, Agilent Technologies, Santa Clara, CA, USA) with a 160 GSa/s sampling rate is used to capture the signals for offline digital signal processing (DSP). As shown in Figure 5, the offline DSP of the FSO signal includes the constant modulus algorithm (CMA), frequency offset estimation (FOE), common phase error (CPE) correction, and decision-directed least mean square (DD-LMS) adaptation.

For the RF receiver, the signal is initially received by a horn antenna (HA) with a gain of 25 dBi. A low-noise amplifier (LNA) with a gain of 20 dB is used to boost the signal before down-conversion. The down-conversion is achieved using a mixer driven by a 75 GHz local oscillator, resulting in an intermediate frequency (IF) of 16.25 GHz. The signal is then amplified by an electrical amplifier (model AT-LNA-0043-3504 from AT Microwave, gain of 35 dB) and captured by a 100 GSa/s oscilloscope (model DSA71604, Tektronix, Beaverton, OR, USA) for subsequent offline digital signal processing (DSP). The offline DSP of the RF signal includes digital down-conversion, CMA, FOE, CPE, and DD-LMS.

## 4. Results and Discussion

**Figure 6:**
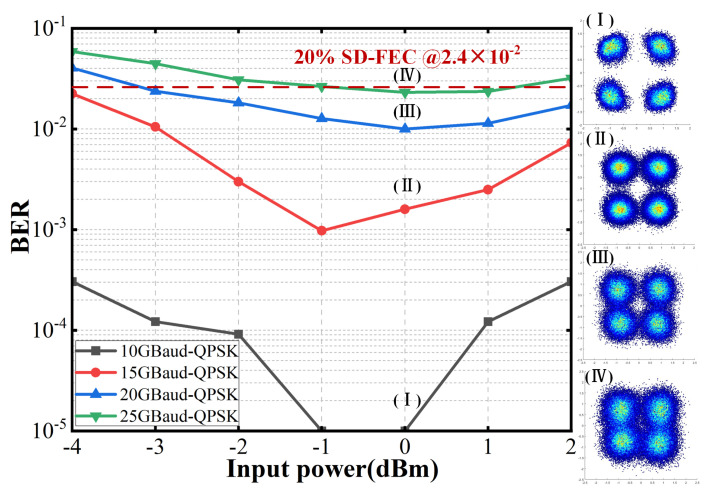
BER of the received RF signals versus the power into the PD.

To evaluate the performance of the hybrid RF/FSO system, experiments were conducted over a 1 m wireless transmission distance. For the RF link, Figure 6 illustrates the bit error rate (BER) curves for QPSK signals at baud rates of 10 GBaud, 15 GBaud, 20 GBaud, and 25 GBaud, with input power to the photodiode (PD) ranging from −4 to 2 dBm. The measured BER values satisfied the 20% soft-decision forward error correction (SD-FEC) threshold of 2.4 × 10^−2^. The BER initially decreased with increasing input power but began to rise beyond approximately 0 dBm, attributed to signal saturation over the short transmission distance, which degraded signal quality. For the FSO link, no errors were observed at the tested baud rates and input optical powers. Consequently, the hybrid RF/FSO system successfully supported 25 GBaud QPSK fusion transmission over the 1 m wireless link.

**Figure 7:**
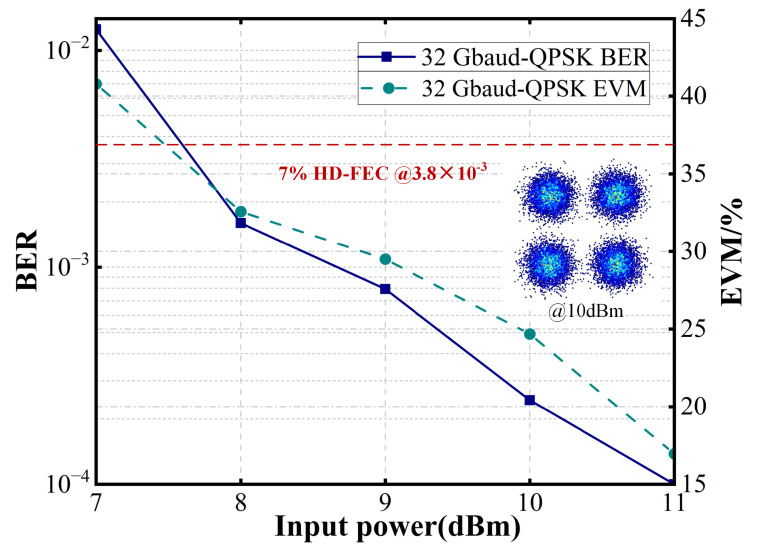
BER and EVM of the received 32 GBaud QPSK FSO signals.

**Figure 8:**
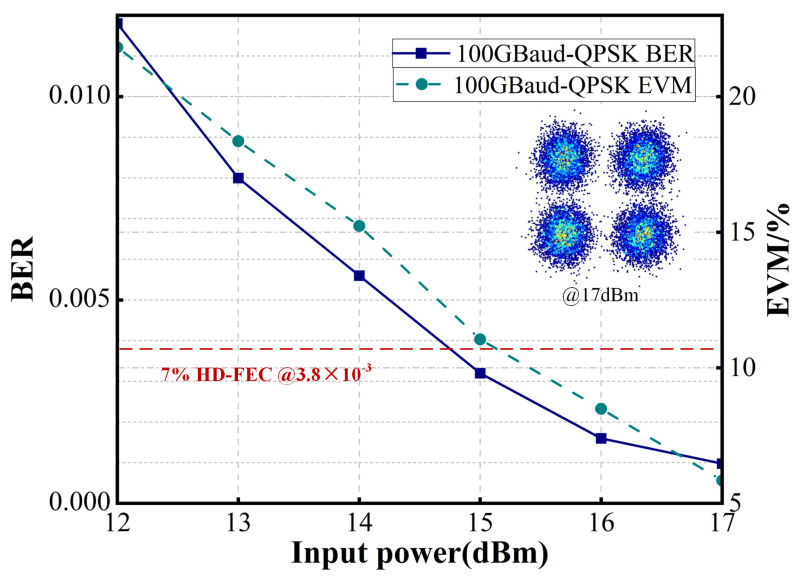
BER and EVM of the received 100 GBaud QPSK FSO signals.

**Figure 9:**
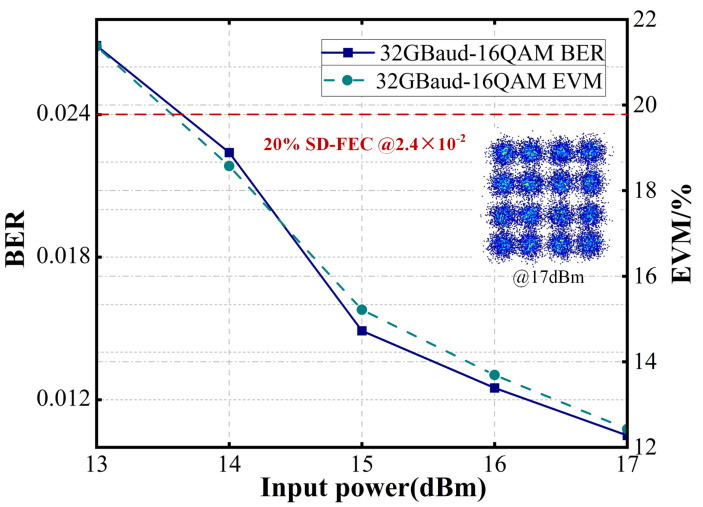
BER and EVM of the received 32 GBaud 16QAM FSO signals.

To further evaluate the system’s performance, the baud rate in the FSO link was increased. As the BER and error vector magnitude (EVM) shown in Figure 7 and Figure 8, the FSO link supports 32 GBaud QPSK transmission when the optical power input to the GRIN lens exceeds 7 dBm, meeting the 7% hard-decision forward error correction (HD-FEC) threshold of 3.8 × 10^−3^. Additionally, at input optical powers above 12 dBm, the system achieves 100 GBaud QPSK transmission while satisfying the 7% HD-FEC threshold of 3.8 × 10^−3^. Furthermore, as illustrated in Figure 9, the system supports 32 GBaud 16QAM transmission at input powers exceeding 13 dBm, meeting the 20% SD-FEC threshold of 2.4 × 10^−2^.

The experimental results demonstrate that the fusion system can support 25 GBaud hybrid RF/FSO transmission, and the FSO link supports a higher baud rate transmission compared to the RF link, as shown in Table 1. This is attributed to the narrower bandwidth of 35 GHz in the W-band electrical signal used for the RF link, whereas the FSO link leverages a spectral range spanning tens to hundreds of RF, offering a significantly broader bandwidth. Consequently, FSO supports higher data rates. Furthermore, as the experiment was conducted indoors over short distances, the optical signal avoided degradation from atmospheric absorption, scattering, and turbulence, maintaining its strength and quality. However, achieving precise alignment for the FSO link is challenging. Misalignment in components such as the collimating lens, dielectric plate, or FSO receiver can cause signal losses. Although RF transmission also requires alignment, it is relatively easy and stable. Outdoors, FSO performance is further affected by ambient light and atmospheric turbulence, while RF transmission is less affected by these factors. Based on the gamma–gamma turbulence and Nakagami-m fading models, theory suggests that the BER of the pure FSO link will exceed the threshold when the fog fading is greater than 250 dB/km or the lateral offset is greater than 1.5 mrad, whereas the hybrid system maintains the reliability by automatically switching to the RF link. This suggests that channel conditions and alignment errors can significantly degrade pure FSO performance, while the hybrid architecture circumvents this risk through dynamic link selection. While the current experiments focus on proof-of-principle in ideal environments, the theoretical model provides a basis for practical analysis, and subsequent work will focus on outdoor field tests to quantify the effects of turbulence, haze, rain, and snow on the hybrid link BER.

In conclusion, the proposed hybrid RF/FSO system establishes an optimal balance between transmission performance and practical deployment efficiency through a unified architecture that integrates optical and radio-frequency signal generation. This innovative co-design eliminates hardware redundancies inherent in conventional implementations, such as separate modulators and antennas typically required in dual-transmission systems, while preserving the distinct advantages of both technologies. The FSO subsystem maintains its high-bandwidth capability, whereas the RF subsystem provides weather-resilient operation essential for uninterrupted service. The architecture enables autonomous adaptation to dynamic environmental conditions: under clear atmospheric conditions, the FSO link delivers peak bandwidth performance, whereas the RF link ensures reliable baseline connectivity during adverse weather conditions such as fog or turbulence, thereby eliminating the alignment and synchronization complexities inherent in dual-transmitter architectures. Through component minimization and utilization of shared propagation pathways, the system provides a compact and energy-efficient solution suitable for applications requiring simultaneous ultra-high data rates and operational robustness, including satellite communication links and mobile network infrastructure deployments. This synergistic integration advances hybrid wireless transmission systems by addressing critical challenges in hardware complexity and environmental adaptability.

## 5. Conclusions

This paper demonstrates the first prototype of a hybrid RF/FSO transmission system with a shared transmitter and transmission link. The system is based on the design of the dielectric board and the properties of light and millimeter waves, and uses photonics-assisted signal generation to enable the FSO and the RF link to share a single transmitter and communication link. In this system, the RF link is used as a complement to the FSO link, which greatly improves the stability and environmental adaptability of the system and reduces the complexity of such hybrid transmission systems by sharing the transmitter design. Experimental results show that the system can support 50 GBit/s hybrid RF/FSO transmission, satisfying the 20% soft-decision forward error correction (SD-FEC) threshold of 2.4 × 10^−2^, and can individually support 100 GBaud QPSK and 32 GBaud 16QAM transmission for the FSO link. The proposed shared transmitter architecture introduces a novel technical framework for wireless communication in dynamic environments. Its optimized hardware integration provides significant advantages in space and power-constrained scenarios, such as satellite relays and mobile backhaul assisted by unmanned aerial vehicles (UAVs). However, current research is primarily confined to laboratory validation, lacking comprehensive assessment of transmission performance degradation under extreme environmental conditions, including atmospheric turbulence, pointing errors, and adverse weather phenomena such as dense fog or heavy rainfall. Future research will focus on long-distance outdoor field trials to systematically evaluate system performance across diverse atmospheric conditions, facilitating its practical implementation in 5G/6G networks. This hybrid structure has the potential to offer high-speed, long-distance, and reliable communications in all environments for future networks.

## Figures and Tables

**Table 1:** Results of the system performance.

Modem Format	RF Link Transmission Rate (bit/s)	FSO Link Transmission Rate (bit/s)
QPSK	50 G	200 G
16QAM	Invalidate	128 G

## Data Availability

The raw/processed data required to reproduce these findings cannot be shared at this time, as the data form part of another ongoing study.
